# Pericoronary fat attenuation index—a new imaging biomarker and its diagnostic and prognostic utility: a systematic review and meta-analysis

**DOI:** 10.1093/ehjci/jeac174

**Published:** 2022-09-07

**Authors:** Marios Sagris, Alexios S Antonopoulos, Spiridon Simantiris, Evangelos Oikonomou, Gerasimos Siasos, Konstantinos Tsioufis, Dimitris Tousoulis

**Affiliations:** First Cardiology Clinic, School of Medicine, ‘Hippokration’ General Hospital, National and Kapodistrian University of Athens, Vas. Sofias 114, 11527 Athens, Greece; First Cardiology Clinic, School of Medicine, ‘Hippokration’ General Hospital, National and Kapodistrian University of Athens, Vas. Sofias 114, 11527 Athens, Greece; Centre for Clinical, Experimental Surgery & Translational Research, Biomedical Research Foundation Academy of Athens, 4 Soranou Ephessiou, 115 27 Athens, Greece; First Cardiology Clinic, School of Medicine, ‘Hippokration’ General Hospital, National and Kapodistrian University of Athens, Vas. Sofias 114, 11527 Athens, Greece; First Cardiology Clinic, School of Medicine, ‘Hippokration’ General Hospital, National and Kapodistrian University of Athens, Vas. Sofias 114, 11527 Athens, Greece; First Cardiology Clinic, School of Medicine, ‘Hippokration’ General Hospital, National and Kapodistrian University of Athens, Vas. Sofias 114, 11527 Athens, Greece; Harvard Medical School, Brigham and Women’s Hospital, 75 Francis St, Boston, MA 02115, USA; First Cardiology Clinic, School of Medicine, ‘Hippokration’ General Hospital, National and Kapodistrian University of Athens, Vas. Sofias 114, 11527 Athens, Greece; First Cardiology Clinic, School of Medicine, ‘Hippokration’ General Hospital, National and Kapodistrian University of Athens, Vas. Sofias 114, 11527 Athens, Greece

**Keywords:** computed tomography, imaging, fat attenuation index, FAI, prevention, unstable plaques, major adverse cardiovascular events

## Abstract

Pericoronary fat attenuation index (FAI) on coronary computed tomography angiography imaging has been proposed as a novel marker of coronary vascular inflammation with prognostic value for major cardiovascular events. To date, there is no systematic review of the published literature and no meta-analysed data of previously published results. We performed a systematic review and meta-analysis according to the Preferred Reporting Items for Systematic reviews and Meta-Analyses guidelines. We systematically explored published literature in MEDLINE (PubMed) before 20 January 2022 for studies assessing FAI in both diagnostic and prognostic clinical settings in patients with or without cardiovascular disease. The primary outcome was the mean difference in FAI attenuation between stable and unstable coronary plaques. The secondary outcome was the hazard ratio (HR) of high FAI values for future cardiovascular events. We calculated *I*^2^ to test heterogeneity. We used random-effects modelling for the meta-analyses to assess the primary and secondary outcomes. This study is registered with PROSPERO (CRD42021229491). In total, 20 studies referred in a total of 7797 patients were included in this systematic review, while nine studies were used for the meta-analysis. FAI was significantly higher in unstable compared with stable plaques with a mean difference of 4.50 Hounsfield units [95% confidence interval (CI): 1.10–7.89, *I*^2^ = 88%] among 902 patients. Higher pericoronary FAI values offered incremental prognostic value for major adverse cardiovascular events (MACEs) in studies with prospective follow-up (HR = 3.29, 95% CI: 1.88–5.76, *I*^2^ = 75%) among 6335 patients. Pericoronary FAI seems to be a promising imaging biomarker that can be used for the detection of coronary inflammation, possibly to discriminate between stable and unstable plaques, and inform on the prognosis for future MACE. Further validation of these findings and exploration of the cost-effectiveness of the method before implementation in clinical practice are needed.

## Graphical Abstract

**Graphical Abstract:**
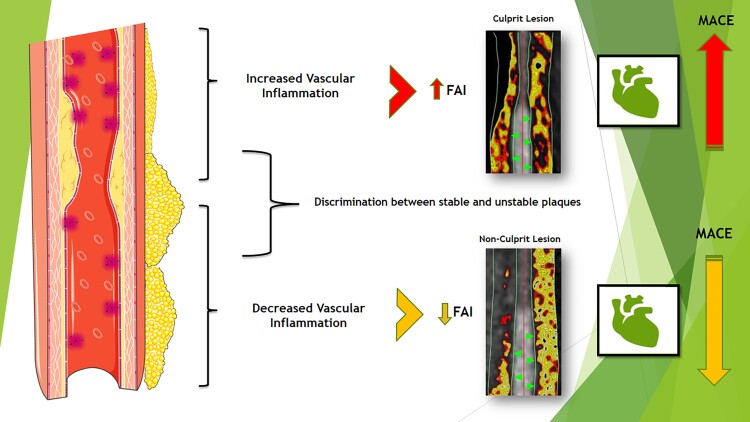

**See the editorial comment for this article ‘Pericoronary adipose tissue attenuation: diagnostic and prognostic implications’, by P. van der Bijl *et al.*, https://doi.org/10.1093/ehjci/jeac175.**

## Introduction

Coronary artery disease (CAD) is an atherosclerotic cardiovascular disease widely affecting people and healthcare systems.^1^ In 2020, American Heart Association released an updated report of Heart Disease and Stroke Statistics, presenting that in the USA, 15.5 million people above 20 years of age suffer from CAD.^2^ Clinicians often face common manifestations of the disease such as stable angina, unstable angina, myocardial infarction (MI), or sudden cardiac death.^3^

The main contributors in plaque formation and atherosclerosis development are endothelial injury, abnormal lipid metabolism, and haemodynamic damage accompanied by flow-mediated inflammatory changes in the endothelium.^3–5^ Atherosclerosis is progressive, leading to atherosclerotic plaque formation in vessels through complex pathophysiological pathways—mainly via inflammatory cytokines.^6,7^ Epicardial adipose tissue and the secreted cytokines have been widely studied as potential contributors to coronary artery pathological characteristics.^8^ Adipocytokines boost the local vascular inflammation, performing the differentiation of the small pre-adipocytes to large ones, with rich intracellular lipid droplets.^8,9^

CAD management constitutes a devastating expenditure for the healthcare system concerning not only treatment but also screening.^10^ Coronary computed tomography angiography (CCTA) is a highly sensitive method for the comprehensive evaluation of plaque characteristics and coronary calcification.^11^ Low-attenuation plaque, positive remodelling, napkin-ring sign, and spotty calcification are found to be independent predictors of major adverse cardiovascular events (MACEs) and plaque rupture.^11,12^ It has been revealed that the coronary artery wall is linked with its coronary perivascular adipose tissue (PVAT) structure via the secretion of inflammatory cytokines. The release of pro-inflammatory molecules from the diseased vascular wall inhibits differentiation and lipid accumulation in coronary PVAT pre-adipocytes in the presence of vascular inflammation. As such, the PVAT of inflamed artery is characterized by low lipid content and an increase in the balance of the aqueous: lipid phase of the tissue. A new CCTA-derived imaging biomarker, the perivascular fat attenuation index (FAI) can trace such phenotypic changes in PVAT and serve as a sensor of vascular inflammation, by detecting respective gradients in PVAT attenuation.^13,14^ Since the generally established computed tomography (CT) attenuation for adipose tissue ranges from −190 to −30 Hounsfield units (HUs), the FAI of inflamed coronary arteries is shifted from more negative (near to −190 HU) to less negative (closer to −30 HU) values. Latest studies showed that higher pericoronary FAI as quantified by CCTA is associated with vascular inflammation and increased risk of cardiac mortality.^13,14^

Our systematic review and meta-analysis aimed to synthesize available evidence on (i) the diagnostic value of pericoronary FAI to classify between stable and unstable coronary plaques and (ii) its prognostic value for MACEs.

## Methods

### Eligibility criteria and study selection

**Figure 1:**
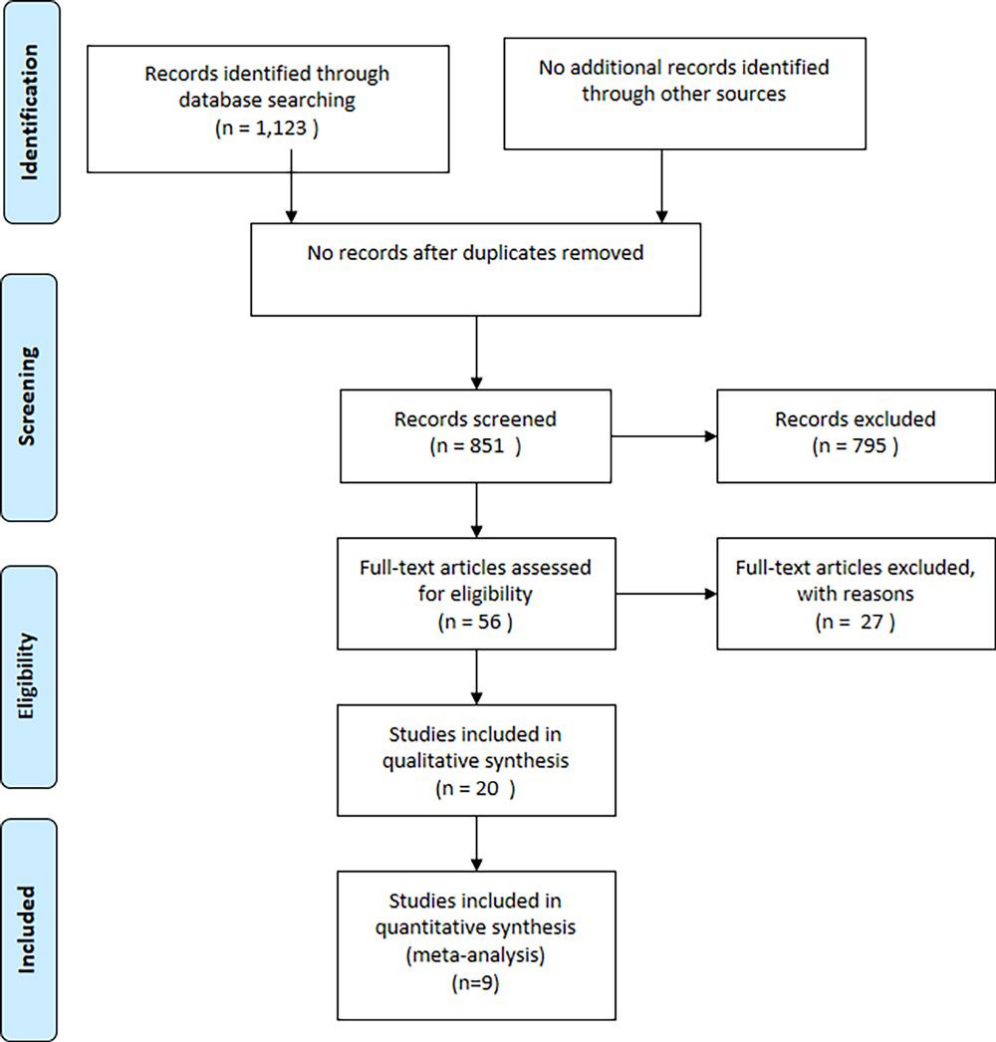
PRISMA flow diagram.

This systematic review and meta-analysis was performed according to the Preferred Reporting Items for Systematic reviews and Meta-Analyses (PRISMA) guidelines^15^ (*Figure 1*). Literature searches were conducted in PubMed until 20 January 2022 with the following algorithm: [‘Tomography, X-Ray Computed’ (Mesh) OR ‘Tomography, X-Ray Computed’ (TW) OR ‘Multidetector Computed Tomography’ (Mesh) OR ‘Multidetector Computed Tomography’ (tw) OR ‘Computed Tomography Angiography’ (Mesh) OR ‘Computed Tomography Angiography’ (tw) OR ‘CCTA’ (tw) OR ‘coronary computed tomography angiography’ (tw) OR ‘coronary CT angiography’ (tw)] AND (‘perivascular’ OR ‘pericoronary’) AND (‘adipose tissue’ OR ‘fat’). Systematic searches were conducted in PubMed/Medline, by two independent investigators, blind to each other, and any disagreements or discrepancies were resolved by consensus with a third investigator.

A study was included in this meta-analysis if it fulfilled the following predefined inclusion PICOTS criteria (*Table 1*):

- Types of studies: prospective clinical cohorts or registries, case–control studies (in English language).
- Types of participants: stable patients with or without CAD; unstable patients, e.g. patients with acute MI, severe valvular heart disease, acute heart failure.
- Types of outcome: changes in FAI value, cardiovascular events, discrimination of stable vs. unstable plaques.
- Time definition: no time constraints on the duration of follow-up period. When duplicates were identified, the most recent study was included unless the earlier version reported more relevant outcomes. Case reports or case series with <10 cases were excluded.

**Table 1:** Abbreviations and PICOTS criteria definition

Abbreviations	PICOTS criteria
ACS, acute coronary syndrome	*P* = Population refers to the sample of subjects you wish to recruit for your study. There may be a fine balance between defining a sample that is most likely to respond to your intervention (e.g. no co-morbidity) and one that can be generalized to patients that are likely to be seen in actual practice
AMI, acute myocardial infarction	
AUC, area under the curve	
CAD, coronary artery disease	
CCS, coronary calcium scoring	
CCTA, coronary computed tomography angiography	
CFR, coronary flow reserve	
CI, confidence interval	
FAI, fat attenuation index	
FFR, fractional flow reserve	
HU, Hounsfield unit	
HR, hazard ratio	*I* = Intervention refers to the treatment that will be provided to subjects enrolled in your study
LAD, left anterior descending artery	
LCx, left circumflex artery	
MACEs, major adverse cardiovascular events	*C* = Comparison identifies what you plan on using as a reference group to compare with your treatment intervention. Many study designs refer to this as the control group. If an existing treatment is considered the ‘gold standard’, then this should be the comparison group
MI, myocardial infarction	
MINOCAs, myocardial infarction with non-obstructive coronary arteries	
NOS, Newcastle–Ottawa Scale	
NCP, non-calcified plaque	
PET, positron emission tomography	
PVAT, perivascular adipose tissue	*O* = Outcome represents what result you plan on measuring to examine the effectiveness of your intervention. There are, typically, a multitude of outcome tools available for different clinical populations, each having strengths and weaknesses
RCA, right coronary artery	
18F-NaF, 18F-sodium fluoride	
	*T* = Time describes the duration for your data collection

### Analysis of coronary PVAT/FAI

Coronary PVAT was measured using CCTA in 3D layers, advancing radially outwards in 1 mm increments from the outer vessel wall. Coronary PVAT attenuation was defined as the average CCTA attenuation in HU of the adipose tissue inside the designated volume of interest, while adipose tissue was defined as all voxels having attenuation between −190 and −30 HU. In one of the studies, Balcer *et al*.^16^ assessed coronary PVAT using non-contrast CT scans. The segmentation of PVAT was done manually. Given the excellent reproducibility of the measurements [intraclass correlation coefficient: 0.95, 95% confidence interval (CI): 0.90–0.97, *P* < 0.001] and for the purposes of completion, we decided to include it in the systematic review even though FAI measurements are not validated in non-contrast scans.

Pericoronary FAI, a novel method for assessing coronary inflammation by analysing routine CCTA, captures changes in PVAT composition driven by inflammatory signals coming from the inflamed coronary artery, by analysing the 3D gradients of perivascular tissue attenuation, followed by adjustments for technical, anatomical, and biological factors. Perivascular FAI was defined as the weighted mean attenuation of all adipose tissue-containing voxels (−190 to −30 HU) lying within a radial distance from the outer vessel wall equal to the diameter of the relevant vessel around the coronary vessels. To avoid the effects of the aortic wall, the most proximal 10 mm segment was excluded as well as the proximal 10–50 mm of the coronary vessel in most of the studies. The proximal 40 mm segment of the left anterior descending coronary artery (LAD), the left circumflex coronary artery (LCx), and the right coronary artery (RCA) were manually traced. RCA segments were used for this meta-analysis since they have been linked with subclinical atherosclerosis and coronary inflammation in previous studies.

### Data extraction and statistical analysis

Two experienced reviewers independently and blind to each other extracted the relevant data from the eligible studies and the final decision was reached by consensus. The objective of our study was to systematically review published studies on PVAT CT attenuation and to collect data from the current literature to perform a meta-analysis for the ability of coronary PVAT attenuation to (i) discriminate between stable and unstable plaques and (ii) predict future MACEs. We used the definition of coronary PVAT as provided by each individual study (*Tables 2* and *3*).

**Table 2:** Characteristics of the included studies

First author, year	Country	Study design	Endpoint	Total patients	Males	Mean age	Results
Antonopoulos 2017^13^	UK	Case–control	FAI—relationship with coronary atherosclerosis	453	366	66.8 ± 0.49	FAI was positively correlated with CAD and CAD extent independently of coronary calcium scoring value, age, gender, and risk factors
Marwan 2017^17^	Germany	Case–control	FAI in atherosclerotic coronary segments	29	22	59 ± 10	Atheromatous coronary segments had higher perivascular FAI compared with normal coronary segments
Balcer 2018^16^	Germany	Case–control	PVAT volume in culprit lesions	46	33	64.4 ± 16.4	In patients with acute myocardial infarction, PVAT volume is strongly and independently associated with culprit lesions in the underlying coronary segments
Goeller 2018^18^	USA	Case–control	FAI—relationship with plaque progression	35	30	59.5 ± 11.3	Baseline high FAI value was positively associated with an increase in non-calcified plaque and total plaque burden
Oikonomou 2018^14^	UK	Prospective cohort	Prognostic value of FAI	3912	2304	—	FAI independently predicts cardiac mortality and non-fatal MI
Dai 2020^19^	China	Case–control	FAI—effects of statins	199	131	69.3 ± 10.4	FAI decreased by statin treatment in a follow-up CCTA scan
Kwiecinski 2019^20^	USA	Case–control	Association of FAI with 18NaF uptake	41	28	65 ± 6	In patients with HRP features on CCTA, increased density of PVAT was associated with focal 18F-NaF PET uptake
Goeller 2019^21^	USA	Case–control	Unstable plaques	111	86	59.2 ± 4.1	Culprit lesions had higher FAI values
Elnabawi 2019^22^	UK	Prospective cohort	Effects of biologic therapy	134	84	51.1 ± 12.1	Biologic therapy for moderate to severe psoriasis reduced perivascular FAI in follow-up CCTA
Gaibazzi 2019^23^	USA	Case–control	Coronary inflammation in patients with MINOCAs or Takotsubo syndrome	212	98	—	Higher FAI value in MINOCA patients
Oikonomou 2019^24^	UK	Prospective cohort	Prognostic value of FAI	1575	—	—	FRP (of PVAT vascularity, inflammation, and fibrosis) independently predicts cardiovascular events
Yu 2020^25^	China	Case–control	FAI relationship with luminal stenosis	167	121	61.8 ± 10.57	FAI was higher around flow-limiting lesions
Hoshino 2020^26^	Japan	Case–control	FAI relationship with luminal stenosis	187	—	—	FAI was higher around flow-limiting lesions
Sugiyama 2020^27^	Japan	Case–control	FAI relationship with unstable (culprit) plaques	540	407	68 ± 7	It is the only study which found no significant difference in FAI between culprit and non-culprit lesions in ACS patients
Yu 2020^25^	China	Prospective cohort	FAI—effects of statin treatment	108	76	67.7 ± 11.1	FAI decreased in a follow-up CCTA of patients who started statin treatment after a baseline CCTA
Nomura 2020^28^	Brazil	Case–control	FAI relationship with myocardial ischaemia	105	46	60 ± 12	FAI was associated with myocardial perfusion abnormalities by PET
Kanaji 2020^29^	Japan	Case–control	FAI relationship with coronary flow reserve	116	96	65 ± 11	Higher FAI was associated with reduced CFR
Lin 2020^30^	USA	Case–control	Pericoronary FRP in stable CAD patients vs. no CAD	180	154	—	Patients with acute MI have a distinct pericoronary adipose tissue radiomic phenotype compared with patients with stable or no CAD
van Diemen 2021^31^	Holland	Prospective cohort	FAI prognostic value for CV evens	539	297	58.6 ± 9.2	RCA PVAT was of prognostic for events beyond anatomical plaque characteristics and ischaemia
Bengs 2021^32^	Switzerland	Prospective cohort	FAI prognostic value for CV evens	314	—	62.5 ± 10.8	FAI did not predict CV events on top of myocardial ischaemia and CCS

CAD, coronary artery disease; PET, positron emission tomography; CCS, coronary calcium score; CCTA, coronary computed tomography angiography; CFR, coronary flow reserve; FAI, fat attenuation index; FRP, fat radiomic profile; MI, myocardial infarction, MINOCAs, MI with non-obstructive coronary arteries; PVAT, perivascular adipose tissue; RCA, right coronary artery.

**Table 3:** Characteristics of the studies included in meta-analysis

Studies included in the meta-analysis evaluating FAI for unstable plaques				
Study	Unstable plaque definition	Traced segments	Analysed segments	Adipose tissue definition
Balcer *et al*. 2018^16^	If a culprit lesion was observed in invasive coronary angiography, patients were evaluated as Type I MI. If however not obstructive coronary artery disease was observed in coronary angiography, patients were evaluated as Type II MI	Left main = 5 mm proximal to bifurcation, proximal LAD = 5 mm distal from bifurcation, mid LAD = 5 mm distal from origin of the first diagonal branch, proximal LCX = 5 mm distal from bifurcation, mid/distal LCX = 5 mm distal from origin of the first obtuse marginal branch, proximal RCA = 5 mm distal from the ostium, mid RCA = in the middle of the descending part of the RCA	Coronary PVAT surrounding the proximal RCA (10–50 mm).	CT attenuation of all voxels between −195 and −35 HU (thresholds used for the definition of adipose tissue)
Sugiyama *et al*. 2020^27^	Low-density non-CP was defined as plaque with attenuation <30 HU. Plaque burden was quantified as plaque volume 100%/vessel volume for each plaque component	The proximal 40 mm segments of the LAD and left circumflex coronary artery and the proximal 10–50 mm segment of the RCA were traced	Coronary PVAT surrounding the proximal RCA (10–50 mm)	CT attenuation of all voxels between –−190 and −30 HU (thresholds used for the definition of adipose tissue)
Goeller *et al*. 2018^18^	The severity of coronary stenosis was visually estimated, as was the presence of calcifications and subtle changes in lumen contour. They defined high-risk plaque features as positive remodelling, spotty calcification, napkin-ring sign, low-attenuation plaque	The proximal RCA (10–50 mm from RCA ostium)	Coronary PVAT surrounding the proximal RCA (10–50 mm)	CT attenuation of all voxels between −190 and −30 HU (thresholds used for the definition of adipose tissue)
Antonopoulos *et al*. 2017^13^	They defined high-risk plaque features as positive remodelling, spotty calcification, napkin-ring sign, low-attenuation plaque	They traced proximal 40 mm segments of the RCA	Coronary PVAT surrounding the proximal RCA (0–40 mm)	CT attenuation of all voxels between −190 and −30 HU (thresholds used for the definition of adipose tissue)
Gaibazzi *et al*. 2019^23^	High-risk plaque features: positive remodelling, spotty calcification, napkin-ring sign, low-attenuation plaque	They traced proximal 40 mm segments of the three major epicardial coronary vessels (for right coronary artery starting 10 mm distal to the ostium, while for left anterior descending artery and circumflex artery starting normally at the ostium)	Coronary PVAT surrounding the proximal RCA (0–40 mm)	They based on the attenuation histogram of perivascular fat within the range −190 to −30 HU

Studies included in the meta-analysis for MACE				
Study		Traced segments	Analysed segments	Adipose tissue definition
Oikonomou *et al*. 2018^14^	They traced the proximal 40 mm segments of all three major epicardial coronary vessels (RCA, LAD, and left circumflex artery)		Coronary PVAT surrounding the proximal RCA (0–40 mm).	They based on the attenuation histogram of perivascular fat within the range −190 to −30 HU
Oikonomou *et al*. 2019^24^	They traced the proximal 40 mm segments of all three major epicardial coronary vessels (right coronary artery, left anterior descending artery, and left circumflex artery)		Coronary PVAT surrounding the proximal RCA (10–50 mm)	They based on the attenuation histogram of perivascular fat within the range −190 to −30 HU
Bengs *et al*. 2021^32^	The RCA, LAD, and the left main coronary artery were traced for ∼50 mm starting at their origin.		Coronary PVAT surrounding the proximal RCA (10–50 mm)	They based on the attenuation histogram of perivascular fat within the range −190 to −30 HU
van Diemen *et al*. 2021^31^	The RCA, the LAD, and the left main coronary artery were traced for ∼50 mm starting at their origin		Coronary PVAT surrounding the proximal RCA (10–50 mm)	They based on the attenuation histogram of perivascular fat within the range −190 to −30 HU

CAD, coronary artery disease; CT, computed tomography; FAI, perivascular fat attenuation index; HRP, high-risk plaque; LAD, left anterior descending artery; MI, myocardial infarction; PVAT, perivascular adipose tissue; RCA, right coronary artery.

Analyses for each endpoint were separately performed using a random-effects model. Inverse variance weights were used in all cases. *I*^2^ statistics were used to assess the heterogeneity across the studies. *I*^2^ > 75% indicated high heterogeneity.^33^ The cumulative incidence of endpoints and the corresponding 95% CI were estimated. Forest plots were used to graphically display the effect size in each study and the pooled estimates. Funnel plots and Egger regression tests were used to assess publication bias. Regarding the difference in FAI between stable and unstable atherosclerotic plaques, the pooled weighted mean difference was plotted. The contribution of each article was weighed. A random-effects model was applied to account for the differences in study design and method of PVAT CT attenuation measurements employed by each research group. A *P*-value of <0.05 was considered significant. Heterogeneity was assessed with a *χ*^2^ test and *I*^2^ test. *I*^2^ > 75% indicated high heterogeneity.^33^ R statistical package version 3.6.0 (https://www.R-project.org/)^34^ was used for all statistical analysis.

### Quality and risk of bias assessment

Study quality scores were ascertained using the modified Newcastle–Ottawa Scale (NOS) for cohort studies. The NOS has been developed to assess the methodological quality of non-randomized studies. Each study was assigned a maximum of four points for selection of the study population, two points for comparability and three points for assessment of the outcome. The criteria for ascertainment of the points and the allocation of points for each study are given in Supplementary data online, *Tables S1* and *S2*. Risk of bias was assessed by two investigators with the Robins-I tool for non-randomized studies and any discrepancies in quality assessment were resolved via consensus.^35^

### A systematic review

#### Pericoronary FAI in atherosclerosis and stable CAD

Inflammation has been implicated as one of the major pathophysiologic mechanisms in the formation of coronary atherosclerotic plaques in previous studies.^36,37^ Pericoronary FAI can serve as a sensitive and specific metric of the vascular inflammatory burden around major epicardial coronary arteries. In the original study that validated FAI as a biomarker of vascular inflammation, it was observed that FAI values around the RCA were lower in healthy patients free of coronary atherosclerosis (*n* = 117) compared with patients with coronary atherosclerosis (*n* = 149).^13^ Furthermore, pericoronary FAI was correlated to CAD independently of coronary calcium scoring (CCS) value, age, gender, and cardiovascular risk factors as well as the atherosclerotic plaque burden in the RCA. In a subsequent study, coronary PVAT attenuation was positively associated with 18F-sodium fluoride (18F-NaF) uptake around atheromatous coronary lesions. The relation between coronary PVAT attenuation and 18F-NaF uptake as examined by positron emission tomography (PET)/CT was firstly studied in a group of patients who underwent CCTA for clinical indications in whom anatomical high-risk plaque features were identified. Higher coronary PVAT attenuation values were observed around plaques with increased 18F-NaF uptake compared with those with lower uptake (−73 vs. −86 HU).^20^ Marwan *et al*.^17^ compared 20 coronary segments with lipid-rich plaques, 20 coronary segments with fibrous plaques and 20 normal coronary segments as characterized by intravascular ultrasound imaging. Coronary PVAT attenuation values were higher in atheromatous compared with normal coronary segments.

The relationship between pericoronary FAI and the haemodynamic significance of coronary atheromatous plaques has been also explored in several studies. In a cohort of 167 patients with 219 lesions assessed by fractional flow reserve (FFR), higher pericoronary FAI values were observed around haemodynamically significant plaques with FFR ≤0.8. In contrast, high-risk plaque features (low-attenuation plaque, napkin-ring sign, spotty calcification, and positive remodelling) were not correlated to the haemodynamic significance of the lesions.^25^ Although FAI was a poor classifier of haemodynamically significant stenoses with an area under the curve (AUC) of 0.63, it increased the diagnostic performance of the model when added on top of luminal stenosis and total plaque volume.^25^ Similar findings were also observed in the study of Hoshino *et al*.^26^ who studied the association of pericoronary FAI with FFR in LAD lesions of intermediate luminal severity. Higher pericoronary FAI values were observed in plaques with low FFR values. Among coronary lesions with FFR < 0.75, those with pericoronary FAI values above −70.9 HU were associated with a three-fold higher odds ratio of being severely stenotic.^26^

Certainly, these findings should be interpreted with caution. Pericoronary FAI has been developed and validated as a metric of biological processes that are affected by vascular inflammation and not as a surrogate marker of luminal stenosis; however, it is possible that in those studies patients with haemodynamically significant lesions had also higher vascular inflammation levels, which could explain the reported relationship between pericoronary FAI and luminal stenosis severity.^25^

Pericoronary FAI has been also associated with coronary flow reserve (CFR) as measured by PET. The value of FAI in predicting lower CFR was mostly observed in patients with CCS <100 without obstructive CAD.^28^ Interestingly, when adjusted for traditional cardiovascular risk factors, CCS, and obstructive CAD, FAI was still related to CFR as estimated by PET. Higher FAI values in patients with non-obstructive CAD and impaired CFR may be explained by the fact that inflamed coronary arteries present impaired vasodilatory potential.^28^ Pericoronary FAI has been also associated with cardiac magnetic resonance-derived CFR in acute coronary syndrome (ACS) patients undergoing that underwent CCTA before percutaneous coronary intervention. The mean FAI of the three major epicardial vessels was the only significant predictor of CFR 1 month later after revascularization for ACS (AUC = 0.63).^29^

FAI is a marker that can also reflect dynamic changes in the PVAT phenotype.^21^ In the study of Goeller *et al*.,^21^ FAI around the proximal RCA was compared between two serial coronary CCTA scans with a mean interval of 3.4 ± 1.6 years. The change in FAI was positively correlated with the change in non-calcified plaque (NCP) burden. A high baseline FAI value was also independently associated to NCP and total plaque burden increase, indicating that high FAI values are an index of a highly inflammatory process which may be the precursor of plaque formation.^21^ On the other hand, changes in perivascular FAI were not correlated with the calcified plaque burden which may be result of the possibly non-inflammatory composition of calcified plaques.

Initiation of statin treatment after the baseline scan was correlated with a decrease in mean FAI in the follow-up CCTA scan in the study of Goeller *et al*.^21^ Similar results were reported by Dai *et al*.^19^ who reported a decrease in FAI in a follow-up CCTA scan in patients that initiated statin treatment after a baseline CCTA scan. The decrease in pericoronary FAI values was significant around non-calcified and mixed plaques, whereas there was no difference around calcified plaques. These findings are in accordance with previous studies’ results and the concept of the anti-inflammatory pleiotropic effects of statins on the vascular wall.^21,38–40^ In agreement with these findings, initiation of biologic therapy (anti-tumour necrosis factor-α, anti-interleukin-12/23, and anti-interleukin-17) in patients with moderate/severe psoriasis led to a decrease in pericoronary FAI in serial CCTA scans. On the other hand, psoriasis patients that received only topical treatment or phototherapy (which do not have any vascular anti-inflammatory effects) had no change in pericoronary FAI values. Therefore, current clinical evidence suggests that pericoronary FAI may be a useful tool to monitor the effects of anti-inflammatory interventions on the vascular wall.^22^

#### FAI as a biomarker of unstable plaques

The value of FAI in discriminating between stable and unstable atheromatous plaques has been explored in several studies. In the original study of FAI by Antonopoulos *et al*.^13^ pericoronary FAI around culprit lesions in acute MI patients was higher than FAI proximally to the lesion (ΔFAI = 8.76 ± 2.87 HU) indicating a higher inflammatory burden around unstable plaques. That finding was independent of stent implantation in the culprit lesion. In a pooled analysis of all lesions, ΔFAI had an excellent diagnostic value for discriminating between stable and unstable lesions.^13^ A serial follow-up CCTA scan was performed 5 weeks after the index ACS event in a subgroup of the cohort and five stable CAD patients to assess changes in FAI. A significant decrease in FAI was observed around culprit plaques, whereas there was no change around stable plaques. It seems likely that pericoronary FAI values may track changes in the local inflammatory status of a culprit coronary lesion in response to the resolution of vascular inflammation after a plaque rupture event or the initiation of statin treatment.^13^ In contrast to other radiomic signatures of PVAT related to PVAT fibrosis and vascularity content remain unchanged after an acute MI event suggesting that the fat radiomic profile may be useful in detecting permanent changes in PVAT phenotype as a result of vascular disease.^24,30^

Goeller *et al*.^18^ also observed that coronary PVAT attenuation was higher around culprit lesions when compared with non-culprit lesions in the same patients and with highest-grade stenoses in matched controls. Sugiyama *et al*.^27^ compared pericoronary FAI between culprit and non-culprit vessels in ACS patients. Although pericoronary FAI was higher in culprit lesions vs. non-culprit lesions in LAD, this was not the case for RCA lesions. Another study also reported no significant differences in coronary PVAT attenuation between culprit and non-culprit lesions in ACS patients, although this was done in non-contrast CT scans and is not directly comparable with the findings of studies using CCTA.^16^

#### FAI in MI with non-obstructive coronary arteries and Takotsubo syndrome

Only one research group has reported findings on pericoronary FAI in patients with MI with non-obstructive coronary arteries (MINOCAs) and Takotsubo syndrome. In the study of Gaibazzi *et al*.,^23^ pericoronary FAI was compared between 106 patients with MINOCA (63 with no identifiable cause, 17 with suspected coronary artery dissection, and 26 with Takotsubo syndrome) and 106 controls. Pericoronary FAI (averaged for the three major coronary arteries) was statistically different between the two groups (−68.37 ± 8.29 HU in the MINOCA/Takotsubo group vs. −78.03 ± 6.20 HU in the control group). It is likely that higher pericoronary FAI values in the MINOCA group may reflect higher levels of vascular inflammation which is implicated both in the pathophysiology of MINOCA and Takotsubo syndrome.^23^ This hypothesis agrees with the results of other studies in the field that have shown a higher inflammatory status (by biochemical or imaging biomarkers) in the coronary vessels of patients with Takotsubo and vasospastic angina.^41–43^

#### Pericoronary FAI for the prognosis of cardiovascular events

The prognostic value of pericoronary FAI for cardiac and all-cause mortality has been evaluated in the Cardiovascular RISk Prediction using Computed Tomography (CRISP-CT) study.^14^ Pericoronary FAI was measured around all three major coronary arteries. Higher FAI values around RCA and LAD were independently associated with cardiac and all-cause mortality.^14^ Notably, when added to a baseline model FAI provided incremental prognostic value on top of age, sex, traditional risk factors, extent of CAD and high-risk plaque features for both cardiac (Δ_AUC_ = 0.049) and all-cause mortality (Δ_AUC_ = 0.075). The added prognostic value of FAI remained significant even when adjusted for CCS.^14^ Interestingly, when treatment with aspirin and/or statins was recommended after CCTA, FAI lost its predictive value.^14^ This suggests that the cardiovascular risk identified by perivascular FAI may be modifiable by optimal treatment (mainly with statins). Importantly, higher FAI values were also linked to increased risk for non-fatal MI, implying that the information captured by pericoronary FAI are indicative of plaque vulnerability and risk of rupture.^14^ In another study of 543 patients who were referred for a CCTA scan and were observed for a median follow-up of 6.6 years, FAI independently predicted all-cause mortality and non-fatal MI events.^11^ However, only RCA FAI was independently associated with the risk of death and non-fatal MI.^11^ In contrast to the previous studies, Bengs *et al*.^32^ concluded that FAI did not offer incremental prognostic value to CCS. In this study,^32^ 314 stable patients were observed for a median follow-up of 2.7 years after a CCTA which was used to measure FAI around RCA, LAD and left main coronary artery. Only RCA FAI was associated with major acute coronary events and was found to be an independent predictor of MACEs when assessed in a multivariate analysis including cardiovascular risk factors, CCTA and myocardial perfusion imaging findings. However, in contrast to CRISP-CT results, FAI around RCA was no longer an independent predictor when CCS was added in the model.^32,44^ However, the power of this study to detect differences between subgroups may have been limited due to the small number of events.

### Meta-analysis of available evidence

#### Selection of studies

Literature search yielded 1123 studies; 20 studies were included in our systematic review, of which four were used for the first part of our meta-analysis [hazard ratio (HR) of higher FAI values for major cardiovascular events] and five for the second one (mean difference in FAI between stable and unstable plaques). In the total of 20 studies, six were case–control studies, eight were prospective cohort studies, and six were retrospective studies. The characteristics of each study are described in *Tables 2* and *3*. The PRISMA flow-chart for the study is presented in *Figure 1*. The PRISMA checklist^45^ is also provided as a Supplementary data online, Appendix.

#### Quantitative synthesis of studies on the prognostic role of FAI

**Figure 2:**
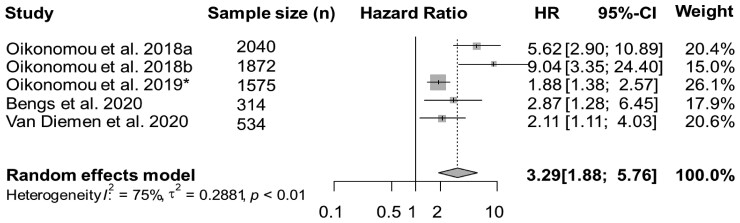
Forest plot for MACE.MACEs, major adverse cardiovascular events; *n*, number; HR, hazard ratio; CI, confidence interval. ^a,b^In the study of Oikonomou *et al*.,^14^ two cohorts from different derivations were analysed. Cohort No. 1 (Erlangen): 1872 subjects, and Cohort No. 2 (Cleveland): 2040 subjects. *The study of Oikonomou *et al*.^24^ included 1575 subjects.

Overall, four studies reported data on MACEs, with a total of 6335 patients. In a meta-analysis of available studies, and by using a random-effects model, FAI was associated with the risk of MACEs (HR = 3.29, 95% CI: 1.88–5.76, *I*^2^ = 75%) (*Figure 2*); however high heterogeneity was observed among studies.

#### Quantitative synthesis on the value of FAI as a biomarker of unstable plaques

**Figure 3:**
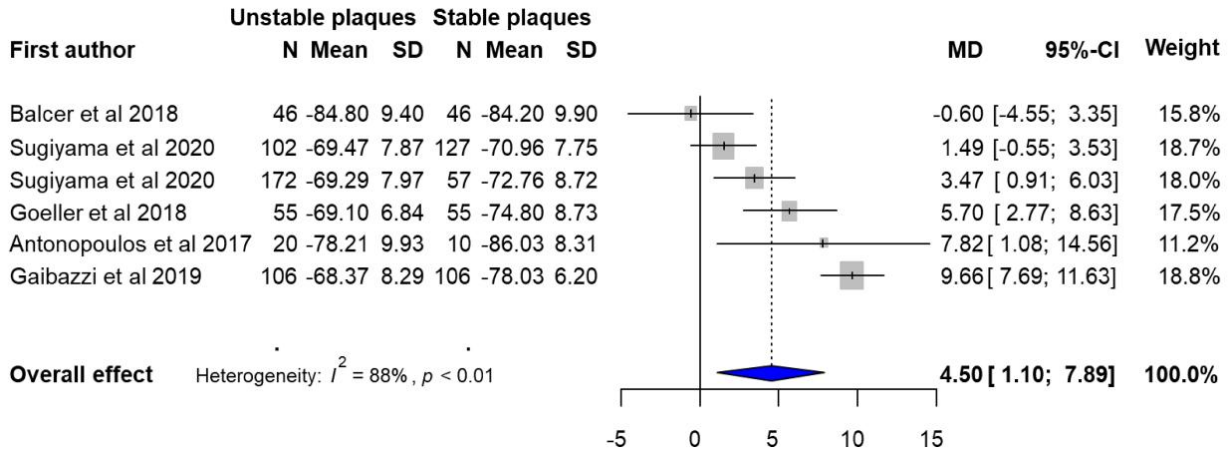
Forest plot of FAI as a biomarker of unstable plaques. FAI, fat attenuation index; *N*, number; MD, mean difference; CI, confidence interval; PVAT, perivascular adipose tissue; LAD, left anterior descending artery; RCA, right coronary artery. Sugiyama provide two separate analyses: one for PVAT in LAD and another for PVAT in RCA.

Overall, five studies compared pericoronary FAI values between stable and unstable plaques, in a total of 902 patients (stable patients *n* = 401, unstable patients *n* = 501). In the quantitative synthesis of available evidence, FAI values were significantly different between stable and unstable coronary plaques (mean difference 4.50, 95% CI: 1.10–7.89, *I*^2^ = 88%) (*Figure 3*), although high heterogeneity was observed among studies.

## Discussion

In the present study, we reviewed published literature to assess the diagnostic and prognostic value of pericoronary FAI. Available evidence suggests that pericoronary FAI is a useful biomarker to detect patients with high levels of vascular inflammation and to identify vulnerable patients at risk for future MACE. In the presented studies, pericoronary FAI values were significantly different between stable and unstable plaques. Given the limited number of studies in the field, there is certainly the need to validate these findings to standardize FAI measurements and explore its diagnostic and prognostic value across a range of pre-clinical probabilities, vendors, and scanner types.

Pericoronary FAI assessment by CCTA provides on the top of coronary anatomy information on the levels of coronary inflammation. The detection of high-grade stenosis lesion is important for angina treatment and revascularization. On the contrary, FAI measurements complement anatomical information derived by standard CCTA with information on the levels of vascular inflammation, which is the main driver of plaque rupture events, and could help in the deployment of preventive strategies. Observations provide the trend that as higher the FAI value is, the more haemodynamically significant is the stenosis, but more research is needed to confirm the assumption.

Pericoronary FAI offered incremental prognostic value for the incidence of cardiovascular events and all-cause mortality. Detection of the residual inflammatory risk could contribute to better risk stratification and discrimination and may lead to application of personalized prevention treatment strategy in patients with highly active inflammatory status in their coronary tree. In addition, FAI may be a useful biomarker to monitor the effects of treatments on vascular inflammation. Interestingly, pericoronary FAI presents a modifiable risk for future MACE as it lost its predictive value when preventive strategies such as statin or aspirin treatment were implemented.^14^ Therefore, pericoronary FAI measurements could be used as a highly specific marker of vascular inflammation (in comparison to circulating plasma biomarkers which are not specific for vascular inflammation) and as an endpoint in future appropriately designed randomized clinical trials to test the effects of novel therapeutics.

### Limitations

Our study has several limitations. First, this was a meta-analysis of observational studies, and thus it should be interpreted in the context of real-world research and its inherent limitations. Secondly, our analysis is based on the meta-analysis of cumulative published data and not on individual patient data. Thirdly, the study design, exact method for FAI analysis, population characteristics, and treatment types differ between studies. Fourthly, the findings on the prognostic value of FAI are based on the results of the CRISP-CT study.

In addition, due to the scarcity of available studies on FAI, it was not possible to perform a meaningful meta-regression analysis based on patients’ baseline characteristics. Also, since perivascular fat density is a continuous measurement via CT scanning analysis and not standardized, it was not possible to provide a binary illustration of FAI derived by the current literature. Finally, CCTA scan quality was heterogeneous between the studies and could possibly affect PVAT attenuation values, and also explain the high statistical heterogeneity that was observed among the included studies.

Review of all available evidence suggests that there is certainly the need to standardize FAI measurements between scanner types and to explore its diagnostic and prognostic value across a range of pre-clinical probabilities. Whether the introduction of FAI in clinical practice is a cost-effective strategy to risk stratify patients and administer preventive treatments remains to be answered by an appropriately designed health economics study.

## Conclusion

We have systematically reviewed published literature for studies on the diagnostic and prognostic value of pericoronary FAI. Available evidence suggests that pericoronary FAI may be a useful biomarker for the detection of unstable coronary plaques, and for the risk stratification of patients for future MACE. Pericoronary FAI could contribute to the identification of vulnerable patients at high cardiovascular risk and help in the deployment of targeted prevention strategies. There is certainly a need for further validation of the findings in larger cohorts providing us more consistent data in order to further expand the use of FAI in clinical practice.

## Supplementary material

Supplementary data are available at *European Heart Journal – Cardiovascular Imaging* online.

## Supplementary Material

jeac174_Supplementary_Data

## Data Availability

The data underlying this article are available in the article and in its Supplementary data online.
