# Supplementary material for: Pericoronary fat attenuation index—a new imaging biomarker and its diagnostic and prognostic utility: a systematic review and meta-analysis
Source: Eur Heart J Cardiovasc Imaging. 2022 Sep 7;23(12):e526–36. doi: 10.1093/ehjci/jeac174 (PMC9840478; doi:10.1093/ehjci/jeac174)
Supplement: jeac174_Supplementary_Data [file jeac174_supplementary_data.zip › Supplementary Tables.pdf]

		right coronary artery starting 10 mm distal to the ostium, while for left anterior descending artery and circumflex artery starting normally at the ostium).		

Studies included in the meta-analysis for MACE

Study	Traced Segments	Analyzed Segments	Adipose Tissue Definition
**Oikonomou et al. 2018 (14)**	They traced the proximal 40-mm segments of all three major epicardial coronary vessels (RCA, LAD, and left circumflex artery)	Coronary PVAT surrounding the proximal RCA (0-40mm).	They based on the attenuation histogram of perivascular fat within the range –190 HU to –30 HU.
**Oikonomou et al. 2019 (34)**	They traced the proximal 40-mm segments of all three major epicardial coronary vessels (right coronary artery, left anterior descending artery, and left circumflex artery)	Coronary PVAT surrounding the proximal RCA (10-50mm).	They based on the attenuation histogram of perivascular fat within the range –190 HU to –30 HU.
**Bengs et al. 2020 (46)**	The RCA, LAD, and the left main coronary artery were traced for approximately 50 mm starting at their origin.	Coronary PVAT surrounding the proximal RCA (10-50mm).	They based on the attenuation histogram of perivascular fat within the range –190 HU to –30 HU.
**Van Diemen et al. 2020 (45)**	The RCA, the LAD, and the left main coronary artery were traced for approximately 50 mm starting at their origin	Coronary PVAT surrounding the proximal RCA (10-50mm).	They based on the attenuation histogram of perivascular fat within the range –190 HU to –30 HU.

Abbreviations: CAD =Coronary artery disease , CT =Computed Tomography, FAI =Perivascular Fat Attenuation Index, HRP =high-risk plaque, LAD =Left anterior descending artery, MI =Myocardial Infarction, PVAT =Perivascular Adipose Tissue, RCA =Right coronary artery

Supplemental Table 1:: Quality assessment of included studies (Prospective Studies) according to Newcastle-Ottawa scale (NOS)

	Selection				Comparability	Outcome			
**Study**	Representativeness of the exposed cohort	Selection of the non-exposed cohort	Ascertainment of exposure	Outcome present at the start	Age/Previous injury	Assessment of outcome	Follow-up duration (>1year)	Adequacy of follow-up of the cohorts	Total

**Bengs et al. (46)**	1	1	1	0	2	1	1	0	7
**Van Diemen et al. (45)**	1	1	1	0	1	1	1	1	7
**Oikonomou et al. (2018) (14)**	1	1	1	0	2	1	1	1	8
**Oikonomou et al. (2019) (34)**	1	1	1	0	2	1	1	1	8
**Elnabawi et al. (33)**	1	1	1	0	2	1	1	1	8
**Dai et al. (2019) (29)**	1	1	1	0	1	1	1	1	7
**Dai et al. (2020) (24)**	1	1	1	0	2	1	1	1	8

Value "0" (in the case the item was not contemplated) or "1" (if the item was contemplated); a maximum score of 2 could be given for the item "comparability."
Studies with 7-9 has high quality, 4-6 high risk, and 0-3 very high risk of bias.

Supplemental Table 2: Supplemental Table 1: Quality assessment of included studies (Case-Control) according to Newcastle-Ottawa scale (NOS)

	Selection				Comparability	Exposure			
**Study**	case definition	cases representativeness	selection of controls	definition of controls	age/ previous injury	ascertainment of exposure	same method of ascertainment	non-response rate	Total
**Marwan et al.** (23)	1	1	1	1	0	1	1	1	7
**Lin et al.** (35)	1	1	1	1	2	1	1	1	9
**Sugiyama et al.** (37)	1	1	1	1	0	1	1	1	7
**Antonopoulos et al.** (13)	1	1	1	1	2	1	1	1	9
**Balcer et al.** (16)	1	1	1	1	2	1	1	1	9
**Goeller et al. (2018)** (36)	1	1	1	1	2	1	1	1	9
**Gaibazzi et al.** (38)	1	1	1	1	1	1	1	1	8
**Nomura et al.** (26)	1	1	1	0	2	1	1	1	8
**Goeller et al. (2019)** (28)	1	1	1	1	1	1	1	0	7
**Yu et al.** (24)	1	1	1	0	1	1	1	1	7
**Hoshino et al.** (25)	1	0	1	1	2	1	1	1	8
**Kanaji et al.** (27)	1	1	1	1	1	1	1	1	8
**Kwiecinski et al.** (22)	1	1	1	1	2	1	1	1	9

Value “0” (in the case the item was not contemplated) or “1” (if the item was contemplated); a maximum score of 2 could be given for the item “comparability.”
Studies with 7-9 has high quality, 4-6 high risk, and 0-3 very high risk of bias.
